# Construction of a Universal Gel Model with Volume Phase Transition

**DOI:** 10.3390/gels6010007

**Published:** 2020-02-27

**Authors:** Gerald S. Manning

**Affiliations:** Department of Chemistry and Chemical Biology, Rutgers University, 123 Bevier Road, Piscataway, NJ 08854-8087, USA; jerrymanning@rcn.com

**Keywords:** gels, volume phase transition, van der Waals, elasticity, polymer network

## Abstract

The physical principle underlying the familiar condensation transition from vapor to liquid is the competition between the energetic tendency to condense owing to attractive forces among molecules of the fluid and the entropic tendency to disperse toward the maximum volume available as limited only by the walls of the container. Van der Waals incorporated this principle into his equation of state and was thus able to explain the discontinuous nature of condensation as the result of instability of intermediate states. The volume phase transition of gels, also discontinuous in its sharpest manifestation, can be understood similarly, as a competition between net free energy attraction of polymer segments and purely entropic dissolution into a maximum allowed volume. Viewed in this way, the gel phase transition would require nothing more to describe it than van der Waals’ original equation of state (with osmotic pressure Π replacing pressure P). But the polymer segments in a gel are networked by cross-links, and a consequent restoring force prevents complete dissolution. Like a solid material, and unlike a van der Waals fluid, a fully swollen gel possesses an intrinsic volume of its own. Although all thermodynamic descriptions of gel behavior contain an elastic component, frequently in the form of Flory-style rubber theory, the resulting isotherms usually have the same general appearance as van der Waals isotherms for fluids, so it is not clear whether the solid-like aspect of gels, that is, their intrinsic volume and shape, adds any fundamental physics to the volume phase transition of gels beyond what van der Waals already knew. To address this question, we have constructed a universal chemical potential for gels that captures the volume transition while containing no quantities specific to any particular gel. In this sense, it is analogous to the van der Waals theory of fluids in its universal form, but although it incorporates the van der Waals universal equation of state, it also contains a network elasticity component, not based on Flory theory but instead on a nonlinear Langevin model, that restricts the radius of a fully swollen spherical gel to a solid-like finite universal value of unity, transitioning to a value less than unity when the gel collapses. A new family of isotherms arises, not present in a preponderately van der Waals analysis, namely, profiles of gel density as a function of location in the gel. There is an abrupt onset of large amplitude density fluctuations in the gel at a critical temperature. Then, at a second critical temperature, the entire swollen gel collapses to a high-density phase.

## 1. Introduction

A gel in its essential form is a networked polymer system, a single “gigantic molecule” [1], immersed in a solvent. Continuing to pursue its essence, we recognize that a gel in its solvent can exist in two distinct states, a dilute phase fully swollen with solvent, and a collapsed, solvent-poor, polymer-rich phase [2]. The dilute phase is analogous to a vapor with the polymer segments playing the role of vapor molecules. The collapsed phase is analogous to a liquid with the dense polymer segments as the liquid molecules. The volume phase transition of a gel is then analogous to the discontinuous condensation of a vapor to its liquid, which was understood by van der Waals as resulting from the instability of intermediate states [3]. It is likely that the volume phase transition of gels is also a result of the instability of intermediate states. It makes sense, then, to draw some inspiration from the van der Waals theory of fluids in any attempt to disclose the physics underlying the gel phase transition.

There is also an essential feature of a swollen gel that is unlike a vapor, namely, the networking of polymer segments by means of cross-links. The segments between cross links cannot diffuse freely up to uniform dispersal limited only by the walls of a container. Instead the tendency toward free diffusion (the concentration of polymer within the gel is greater than the vanishing polymer concentration in the bathing solvent), is checked by a net restoring force originating at the cross links. Attempts to describe the restoring force have traditionally relied on the theory of entropic rubber elasticity. Entropic elasticity may be apt for the collapsed state, where there is little stress on the cross links, and the polymer segments between them are in a flexible conformation. But rubber elasticity is counterintuitive in the fully swollen state of maximum gel volume limited by the networking. The free diffusion as frustrated by cross links results in a state of internal tension, unlike the relaxed internal configurational state of rubber. We need not rely on intuition in this matter, however, as measurements of Poisson’s ratio provide direct experimental evidence that the swollen state of a gel is not analogous to rubber.

Hirotsu [4] has measured Poisson’s ratio σ for a gel, that is, the absolute value of the ratio of transverse to longitudinal strains. Nearly all materials have positive values of σ, meaning that they become thinner when stretched, and thicker when compressed. For rubber, σ is very close to 0.5, the ideal value expected for a material with invariant volume. The value obtained by Hirotsu for the collapsed gel was indeed close to 0.5, like rubber, but σ for the swollen gel was about half this value, more closely corresponding to Poisson ratios of common hard solids such as steel, copper, glass, carbon fiber, and so forth.

One hesitates to critique a body of theoretical literature remarkable for its prediction of the gel phase transition before it was observed in the laboratory. But it is fair to remark that insight is lost when a theory does not distinguish between essential and secondary effects. Another consequence of possibly unnecessary complexity is a trail of dubious concepts, such as a swollen equilibrium state characterized by zero osmotic pressure; the existence of metastable states that place gels outside the purview of the Gibbs phase rule; and perhaps most seriously, as we have just seen, the application of rubber elasticity to a system that has as much in common with steel as with rubber.

The purpose of this paper is to review a recent attempt to reduce the theory of the volume phase transition of gels to its essential underlying physics, while frankly paying homage to van der Waals by building on his insights [5]. This goal is accomplished by addition of a simple representation of the restoring forces in the gel network to the van der Waals equation of state as adapted in a transparent way to binary solutions. Features then appear in the isotherms that go beyond the cubic “wiggle” of the van der Waals curves. We include new material on the bulk modulus, but since this paper is mostly a review, the reader is encouraged to consult the original article [5] for further elaboration. The first five figures are taken wholly or in part from the original open-access article.

## 2. Theory

### 2.1. Van der Waals Universal Equation of State for a Pure Fluid

The universal, or reduced, form of the van der Waals equation of state for a pure one-component fluid is,

(1) $$\left(p+3\varrho^{2}\right)\left(3-\varrho \right)=8\varrho t$$

In this form all quantities are dimensionless. For example, *p* is the ratio of the physical pressure *P* to the value of the pressure at the critical point $P_{c}$. Thus p=1 when the fluid is in its critical state. Similarly, $t=T/T_{c}$, where *T* is the Kelvin temperature, and $T_{c}$ the critical temperature, so that *t* is also equal to unity at the critical point. The number density of the fluid is the number of molecules per unit volume, and ϱ is the ratio of the number density to its value at the critical point, where ϱ=1. For simplicity, we refer to the “renormalized” quantities p,t, and ϱ as pressure, temperature, and density. Notice that the maximum value of density is 3 (closely packed liquid), while for low densities, the equation reduces to p=(8/3)ϱt (dilute gas with negligible interactions, or, simply, an ideal gas).

**Figure 1:**
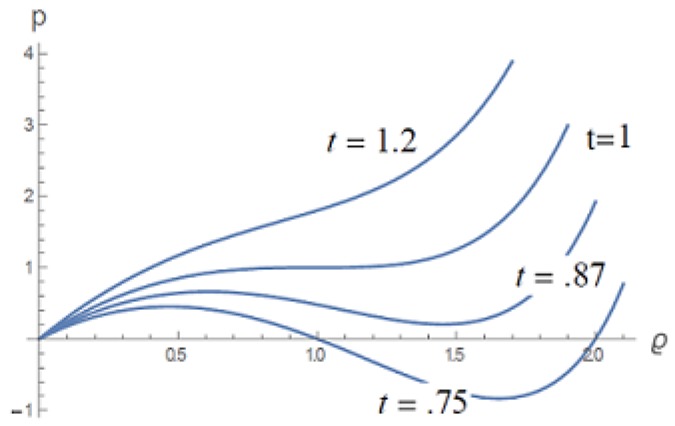
Representative isotherms for the universal (reduced) van der Waals equation of state for a pure fluid. The quantities *p*, *t*, and ϱ are, respectively, the reduced pressure, temperature, and number density [5].

Figure 1 displays four isotherms according to Equation (1), supercritical, critical, and two subcritical isotherms. The supercritical isotherm for t>1 shows the progressive transition from a vapor state at low density to a high-density liquid state. The isotherm for t=1 passes through the critical state of infinite compressibility, ∂p/∂ϱ=0, at p=ϱ=1. For the subcritical isotherms t<1, there are intermediate ranges of densities where the compressibility is negative. Such states are unstable and, if formed, would immediately disappear, yielding to stable coexistence of vapor and liquid phases observed as a discontinuous transition. For these isotherms, a horizontal line intersects liquid and vapor regions of positive compressibility. However, for the “coldest” isotherm shown, at t=0.75, a horizontal line at negative pressure intersects only the liquid portion of the isotherm, indicating that only a liquid can stably exist at negative pressure (i.e., under tension).

### 2.2. Van der Waals Equation of State for a Solution

The universal van der Waals equation is applicable to a pure fluid consisting of identical and independent, albeit interacting, molecules. As such, it cannot serve by itself as a minimal model for the volume transition of a gel, since a minimal gel model must at least recognize, first, that a gel is a two-component, solute/solvent, system; and second, that the gel material (solute) is a networked mesh. The first requirement is easily accomplished. The McMillan–Mayer theory of solute/solvent free solutions is among the few exact statistical mechanical theorems. It states that the virial series for the pressure *P* of a real gas goes over to a virial expansion for the osmotic pressure Π of a solution (the difference between the pressure of the solution and the pressure of pure solvent at equilibrium across a membrane impermeable to solute). The only difference is that the intermolecular forces in vacuum underlying the virial coefficients for the real gas must be replaced by the corresponding solute-solute interactions as they exist in free solution as mediated by solvent. A corollary of the McMillan–Mayer theorem is that any equation of state for a gas has its counterpart as an equation of state for osmotic pressure. An obvious example is the van’t Hoff equation ΠV=NkT as the solution analog to the ideal gas equation of state PV=NkT.

To apply these considerations in the present context, we write a universal van der Waals equation for the osmotic pressure of a free solution in analogy to Equation (1),

(2) $$\left(\pi +3\alpha \varrho^{2}\right)\left(3-\beta \varrho \right)=8\varrho t$$

Here, the dimensionless temperature *t* has unchanged meaning; the dimensionless density ϱ is the number density of the solute; and π is the dimensionless osmotic pressure of the solution. Aside from the analogy between osmotic pressure and pressure, there is another important distinction between Equations (1) and (2). In the van der Waals theory of a pure fluid, the interaction constants *a* (measuring pairwise attraction) and *b* (the excluded volume parameter) are independent of temperature. For a solution the solute–solute interactions are mediated through the random movements of solvent molecules and therefore must depend on temperature. The parameter α in Equation (2) is the ratio $a\left(tT_{c}\right)/a\left(T_{c}\right)$, and $\beta =b\left(tT_{c}\right)/b\left(T_{c}\right)$. Thus α and β are identically equal to unity if *a* and *b* do not depend on temperature, and if they do depend on temperature, they are both equal to unity at the critical point t=1.

**Figure 2:**
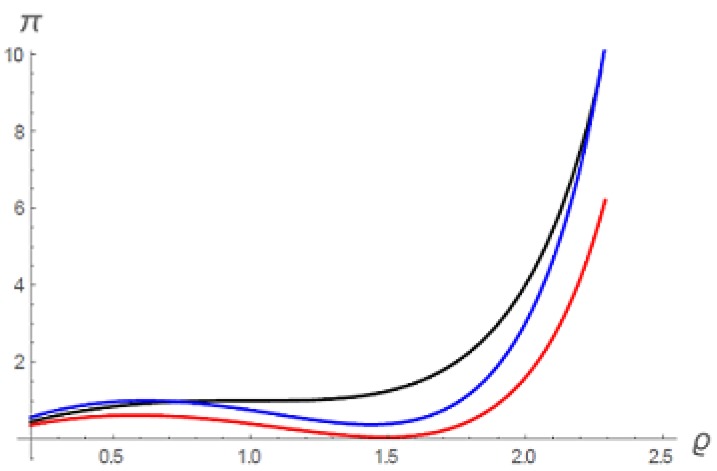
Isotherms for a MacMillan-Mayer Van der Waals solution (Equation (2)). β=1 for all isotherms. Black: t=1, hence α=1. Blue: $\alpha =t^{1.5}$, t=1.3. Red: α=1, t=0.85.

In Figure 2 we show three solution isotherms. We expect the excluded volume parameter to be relatively insensitive to temperature, and so have set β=1 for all three cases. The black curve for t=1 is the same as the critical isotherm in Figure 1, since α=1 when t=1. The red isotherm is for t=0.85, that is, subcritical. For this isotherm, we have assumed α to be insensitive to temperature, so that α=1. This isotherm is therefore very similar to the t=0.87 isotherm in Figure 1. In the unstable range of solute densities, the solution separates into solvent-rich and solvent-lean phases. The blue isotherm is for t=1.3, a supercritical isotherm. It also exhibits an unstable range of solute densities, hence phase separation, because we have taken α as temperature-dependent in generating this isotherm, arbitrarily setting $\alpha =t^{1.5}$ as a numerical example. In other words, we have assumed here that the intermolecular attraction among solute molecules increases with temperature, as might be true, for example, if the solvent is water while the solute is soluble but contains a hydrophobic moiety. In this case the temperature dependence of phase separation is inverted; phase separation occurs at *t* higher than critical. We can conclude that ordinary van der Waals theory, adapted to solutions, provides the basic physics underlying the full range of phase behavior of free polymer solutions.

The effect of solute-solute interactions can be seen by comparing this equation with the form it takes at low density, π=(8/3)ϱt. But a more direct measure of solute-solute interactions is provided by the solute activity coefficient γ, which can be obtained first by solving Equation (2) for π=(8/3)φϱt in the form of a correction from low densities given by the osmotic coefficient factor φ, and then by integrating the Gibbs–Duhem equation, ρdln(γρ)=d(φρ). We find,

(3) $$\ln\gamma =\frac{\beta \varrho }{3-\beta \varrho }+\ln\left(\frac{3}{3-\beta \varrho }\right)-\frac{9\alpha \varrho }{4t}$$

The deviation of the activity coefficient from its value unity in the dilute limit ϱ→0 is a measure of solute-solute interactions. See the original paper [5] for a plot of γ as a function of density at various temperatures for the case α=β=1.

### 2.3. The Networking Energy

**Figure 3:**
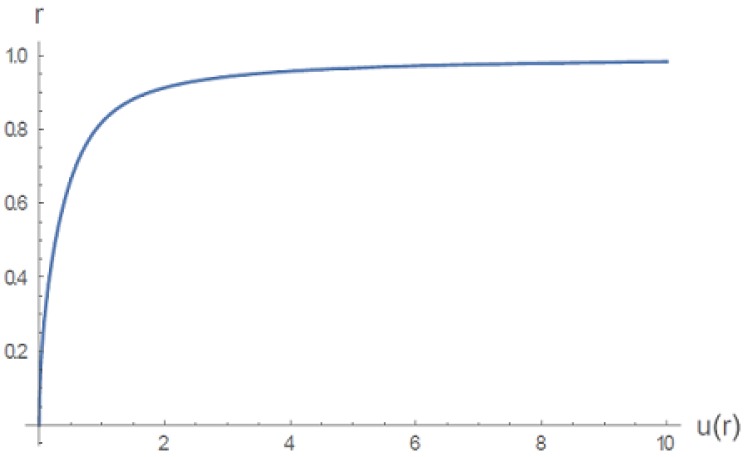
Universal radial distance as the inverse function of network potential energy u(r) in dimensionless units [5].

There remains the second requirement for a minimal gel model, that it include the existential fact of a networked mesh. The gel material is not like a free solution with freely diffusing solute molecules. Free diffusion is checked by the networking, and we make as our second central assumption the idea that the primary physical consequence of the networking is prevention of free diffusion. To implement this idea, we have taken radial symmetry as a simple geometry for the gel, and defined the square of a dimensionless radial distance as $r^{2}=\left(1/2\right)\left(q/k_{B}T\right)R^{2}$, where *R* is physical distance from the center of the spherical gel, $k_{B}T$ is the product of the Boltzmann constant and Kelvin temperature, and *q* is a constant specific to the type of gel material. We then assume that $r^{2}$ is a Langevin function of a dimensionless variable *u*,

(4) $$r^{2}=\coth\left(3u\right)-\frac{1}{3u}$$

where the first term is a hyperbolic cotangent. Close to the gel center, where *r* is small, $u=r^{2}$, so *u* is an elastic energy that nonlinearly approaches infinity near the gel boundary r=1. Figure 3 shows *u* as a function of *r* but in implicit form r(u). It is seen as a restraining elastic energy that reaches infinity at the gel boundary r=1, preventing outward diffusion driven by the infinitely steep gel concentration gradient there (the gel concentration is zero outside the gel). See the original paper [5] for further discussion of why this form for u(r) qualitatively mimics the actual three-dimensional networking constraints of a gel.

### 2.4. The Gel Isopotentials

The local solute chemical potential μ(r) is the ideal vehicle for combining all three effects—the tendency to outward diffusion of the gel material, the inward-acting networking forces restraining diffusion, and solvent-mediated van der Waals interactions—into an equilibrium state,

(5) $$\mu \left(r\right)-\mu^{0}=\ln\varrho \left(r\right)+\ln\gamma \left(r\right)+u\left(r\right)$$

where $\mu^{0}$ is a constant. We have written the chemical potential as dimensionless, in units of $k_{B}T$. The three terms on the right-hand side represent the three effects. The location dependent entropy of mixing term lnϱ(r) expresses the tendency to free outward diffusion, the activity coefficient term accounts for the van der Waals interactions, and the potential energy u(r) resists diffusion. The condition for equilibrium is that the chemical potential must be constant throughout the gel, dμ/dr=0, and integration of this condition produces “isopotential” lines, that is, curves of constant chemical potential μ. The isopotentials are gel density profiles that show how ϱ varies with distance *r* from the gel center.

**Figure 4:**
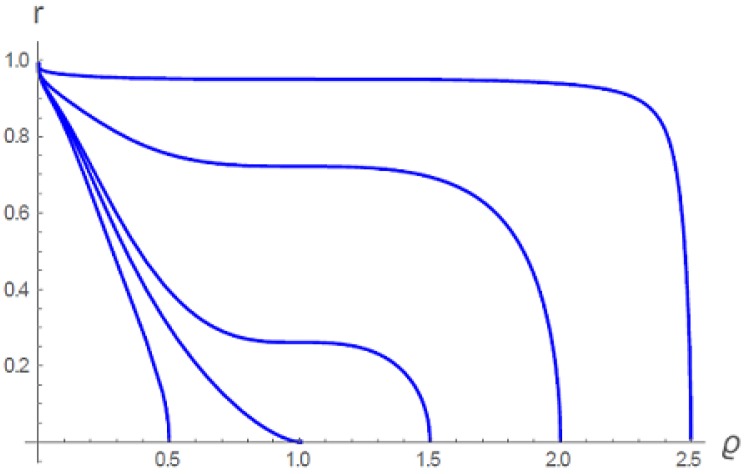
Gel density profiles, all at t=1, but with differing choices of the free parameter $\varrho_{0}$, the density at r=0 [5].

**Figure 5:**
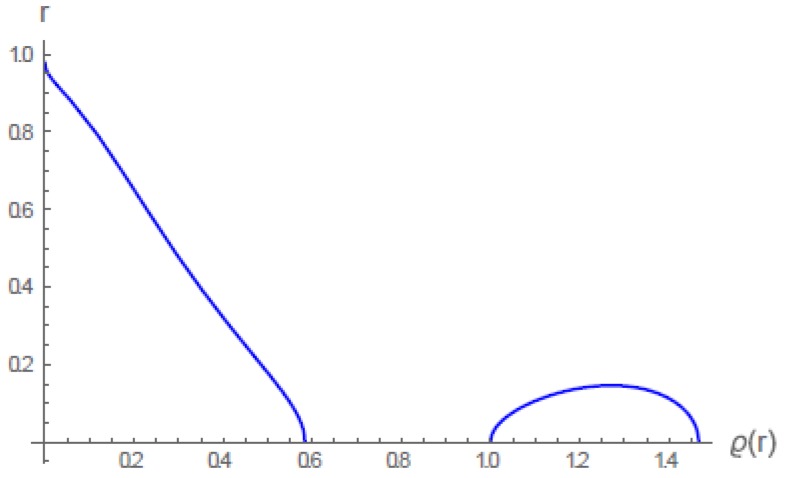
The multivalued isopotential (density profile) at t=0.95 for the single choice of free parameter $\varrho_{0}=1$, which appears as the middle, unstable, value of the density at the origin r=0. The smaller value near 0.58 and the larger value near 1.47 are not chosen as the parameter $\varrho_{0}$; they appear automatically as a consequence of the single choice $\varrho_{0}=1$. The portions of the profile featuring diminishing density as r increases are stable; the left-hand part of the “bulge’’, with density increasing as r increases, is unstable [5].

**Figure 6:**
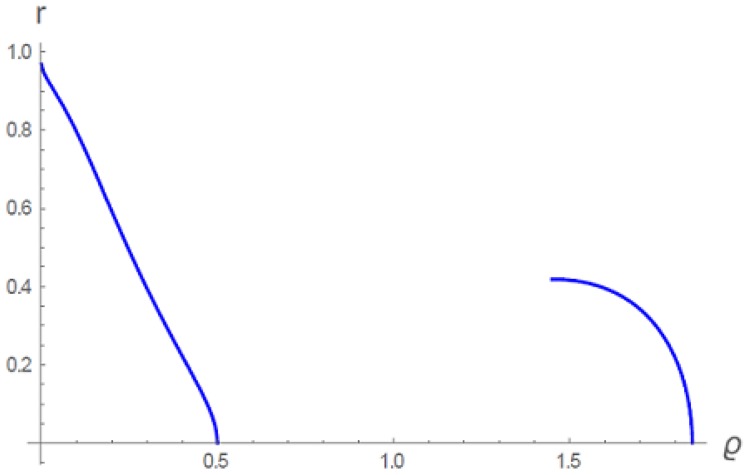
The isopotential (density profile) for t=0.87 and choice of free parameter $\varrho_{0}=0.5$. Only the stable parts of the profile are shown. The low density branch and high density branch contain the same amount of gel material, and respectively represent the swollen and collapsed phases at the transition temperature [5].

In the following series of figures, Figure 4, Figure 5 and Figure 6, we show some examples of these isopotentials, or density profiles (we use these terms interchangeably). Because the Langevin network potential energy *u* is defined from Equation (4) as the inverse function r(u), it turns out that these profiles are displayed as inverse functions $r(\varrho ;\varrho_{0})$, where the free parameter $\varrho_{0}$, the density at r=0, originates as an integration constant. Radial distance *r* is on the vertical axis, density ϱ on the horizontal axis. The figures show numerical results for a “lower critical temperature” gel, i.e., a gel that undergoes its volume phase transition toward collapse when the temperature *t* decreases. In these figures, α=β=1. See Figure 9 of the original paper [5] for numerical analysis of the upper critical temperature case.

All of the curves in Figure 4 were calculated for the same temperature t=1. This value of *t* is critical in the sense that if t≥1, gels of different densities cannot coexist. The different isopotentials in Figure 4 result from different choices of the free parameter $\varrho_{0}$, the density at r=0. For example, the density profile with $\varrho_{0}=2.5$ represents a highly cross-linked gel, so that the gel must have a very high density of gel material even when fully swollen (recall the value 3 as the maximum, i.e., close packing, value of ϱ). In this case the density remains nearly constant up to the periphery r=1, then abruptly falls to zero. For a gel prepared to be dilute when swollen, for example, $\varrho_{0}=0.5$, the density falls off more gradually to zero at r=1. But for each gel (each value of $\varrho_{0}$), there is only a single density profile, representing the swollen gel as extending all the way to the maximum boundary r=1.

When t<1 a new phenomenon appears. In Figure 5 we have set t=0.95. We also chose a single value $\varrho_{0}=1.0$ for the free parameter, so that Figure 5 displays only a single isopotential. But this isopotential is disconnected. It is not single-valued for all values of *r*. It is single-valued only for r>0.15. A horizontal line drawn at any value of *r* greater than 0.15 intersects only one value of the density ϱ. These values of ϱ are relatively small (ranging from 0.52 at r=0.15 to zero at the periphery r=1). The gel is therefore fully swollen (it extends to r=1), and its outer part (r>0.15) is uniformly dilute. Next, we describe the inner part of the gel in Figure 5, that is, r<0.15. A horizontal line drawn for any of these smaller values of *r* intersects the isopotential at three distinct values of the density ϱ. For example, regard the horizontal axis itself, r=0. Even though we chose a single value of the free parameter $\varrho_{0}$, this value, unity, representing density at r=0, appears on the horizontal axis only along with two others, one smaller, the other larger. We have proved in the original paper [5] that the intermediate value ϱ(0)=1 is unstable. The lower and higher ones are stable, ϱ(0)=0.58, and ϱ(0)=1.47. The physical meaning is that at the temperature t=0.95, condensation nuclei appear at the origin r=0. These “liquid” gel nuclei with density 1.47 coexist (fluctuate) with a much more dilute “vapor” gel phase with density 0.58. As another example, take a horizontal line at r=0.15. It intersects the left-most, dilute, branch of the isopotential at ϱ=0.52, and it touches the right-most “bulge” of the density profile at its maximum at ϱ=1.27. In the spherical shell of the gel with radius r=0.15, a “vapor” gel phase with density 0.52 coexists with condensed “liquid” nuclei with density 1.27.

Another consideration is displayed in Figure 6. In this figure, there is a single isopotential at t=0.87 with the free parameter chosen as $\varrho_{0}=0.5$. The isopotential as shown is double-valued, because in the figure we have not drawn the unstable part of the condensation bulge (it exists, as in Figure 5, but it is not drawn in Figure 6). The amount of gel material is the same in both branches, that is, the areas to the left of each branch, normalized for spherical symmetry, are equal. Figure 6 therefore represents the volume phase transition for the gel at t=0.87. The dilute density profile on the left is the swollen gel, and the collapsed high-density gel is on the right. The radius of the swollen gel is r=1, while the radius at the outer periphery of the condensed gel is seen from the figure to be 0.42. The volume of the collapsed phase at the transition temperature is therefore reduced to 7.4 per cent of the swollen volume for a spherical gel. As the temperature is lowered past the transition temperature, the condensed gel contracts further (not shown).

The detailed numerical analysis in the original paper for the choice $\varrho_{0}=0.5$ can be summarized. When t≥0.9443…, the gel is fully swollen up to the maximum periphery r=1. The density is dilute, tapering to zero at r=1. When *t* falls below 0.9443…, nuclei of dense gel material appear. When *t* reaches the value 0.87, the gel volume transition occurs. Figure 6 here shows the density profiles of swollen and collapsed gels in equilibrium at t=0.87. The isopotential colored orange in Figure 8 of the original paper [5] shows density profiles at an intermediate t=0.93, when nuclei of dense gel material are present inside the swollen gel.

### 2.5. The Bulk Modulus

The bulk modulus $K=-V{\left(\partial P/\partial V\right)}_{T}$ is defined as the inverse of the thermodynamic compressibility of the material [6]. Noting that *K* has the units of pressure, we can define a universal modulus $\kappa =K/P_{c}$, and then in terms of universal quantities,

(6) $$\kappa =\varrho {\left(\partial p/\partial \varrho \right)}_{t}$$

For a van der Waals pure fluid, the equation of state Equation (1) can be solved for pressure *p* and straightforwardly differentiated to give,

(7) $$\kappa =-\frac{6\varrho }{{\left(\varrho -3\right)}^{2}}\left[\varrho {\left(\varrho -3\right)}^{2}-4t\right]$$

At the critical point t=ϱ=1, κ vanishes, as can also be seen visually from the isotherm at t=1 in Figure 1.

The bulk modulus of gels has been measured [4,7]. In the neighborhood of the volume phase transition, *K* for the fully swollen gel is an order of magnitude less than for the collapsed phase. We can also obtain this result for our universal gel. We can show that κ for the gel is identical to Equation (7) for a van der Waals fluid with the important exception that for the gel ϱ(r) replaces the uniform value ϱ in Equation (7). In other words, for the gel κ(r) is a local quantity. To compare with experiment, we take an average,

(8) $$<\kappa >=\frac{1}{\left(4\pi /3\right)r_{b}^{3}}\int_{0}^{r_{b}}\kappa \left(r\right)4\pi r^{2}dr$$

with $r_{b}$ the radius of the spherical gel at its periphery. Since *r* can be expressed as a function of ϱ, and κ as a function of ϱ from Equation (7) above,

(9) $$<\kappa >=\left(3/r_{b}^{3}\right)\int_{\varrho (0)}^{\varrho (r_{b})}\kappa \left(\varrho \right)r^{2}\left(\varrho \right)\left(dr/d\varrho \right)d\varrho$$

Numerical evaluation of the integral at the transition temperature t=0.87 (for $\varrho_{0}=0.5$, see Figure 6), results in <κ>=0.17 for the swollen gel, $r_{b}=1$; and for the collapsed phase with $r_{b}=0.42$, <κ>=4.03, an order of magnitude greater.

**Figure 7:**
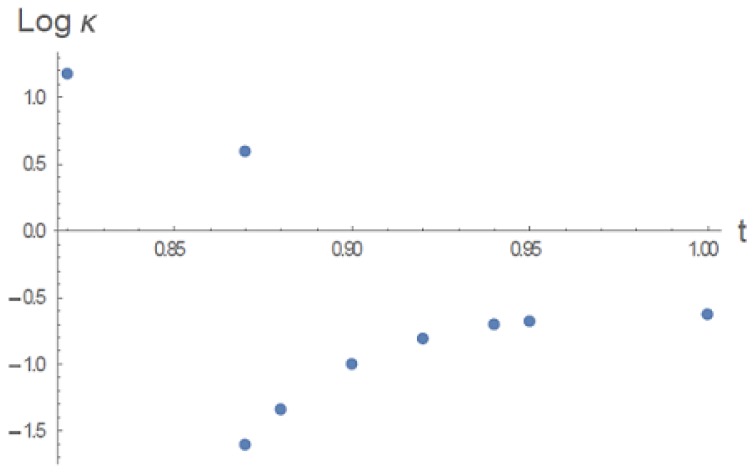
Bulk modulus κ as a function of reduced temperature *t* through the collapse transition at t=0.87, $\varrho_{0}=0.5$.

An interesting aspect of the gel volume transition is the softening of the gel as the transition is approached, as detected by a marked decrease of the bulk modulus [4] (or Young’s modulus [7]). It is expected that density fluctuations are the underlying cause [8,9]. Our universal gel exhibits this behavior, as indicated in Figure 5. For example, at t=0.95, the density does not fluctuate in the outer part of the swollen gel (r>0.15 in Figure 5, where the density is single-valued and dilute, left branch). But for r<0.15, the density fluctuates between low-density and high-density values, as discussed in connection with Figure 5. For a fixed value of *r* in this fluctuating region, the pressure does not change but the density undergoes discrete changes. The modulus κ therefore vanishes in this region (stress is absorbed by the density changes with no resistance). The points in Figure 7 were calculated by taking an average in the range 0.15<r<1 while setting κ=0 for r<0.15.

### 2.6. Gibbs Phase Rule

An equilibrated swollen gel is a binary solution in which the free outward diffusion of solute is prevented by the networking of the solute itself. The mechanical restraint on free outward diffusion creates a balancing pressure gradient that can, if one wishes, be called an osmotic pressure. But it is important, at least for conceptual purposes, not to regard gel swelling as caused by osmotic pressure. The difference between the pressure inside the gel and outside is a consequence, not a cause, of mechanically restrained swelling. One of the dubious concepts sometimes inserted into the theory and even computer simulations of the volume transition of gels is requirement of a negative pressure. Although the t=0.75 van der Waals isotherm in Figure 1 possesses a stable region of negative pressure *p*, the pure liquid to which it applies is not a gel. For the gel, the hydrostatic equation,

(10) $$\frac{dp}{dr}=-\frac{8t}{3}\varrho \frac{du}{dr}$$

implies that the pressure inside an equilibrated gel is always positive (assuming ordinary conditions in the bathing solvent outside the gel, for example, 1 atm) [5].

The pressure difference inside and outside the gel is responsible for failure of the Gibbs phase rule to describe the number of “degrees of freedom” of equilibrated gel systems, but the basic physics underlying the Gibbs rule is still valid, and there is no need to invoke metastable gel states. The case of the three-phase gel system (swollen and collapsed gel coexisting in bathing solvent) is discussed in detail in the original paper [5]. Here we review only the simplest related example, a standard two-phase osmotic system consisting of a solute-solvent solution (one phase) separated from pure solvent (the other phase) by a semi-permeable membrane. In general non-equilibrium conditions, each phase has its own temperature, its own pressure, and the solution phase has its solute concentration, for a total of five non-equilibrium degrees of freedom. At equilibrium, there are two constraints, the equality of temperatures and of solvent chemical potentials. The degrees of freedom at equilibrium are in number, therefore, equal to 5−2=3, and the three equilibrium degrees of freedom are the values of temperature, pressure in the pure solvent phase, and solute concentration in the solution phase. Thus, even though rote application of the Gibbs phase rule gives the wrong answer, 2−2+2=2, the standard osmotic pressure experiment requires no introduction of new considerations.

## 3. Scope of the Model and Open Questions

The author’s motivation in formulating and analyzing a universal gel model in Reference [5] and reviewing it here is his belief that overly complicated theories of the behavior of gels, along with resort to atomistic computer simulations, has resulted in a loss of insight into the basic physical principles governing this interesting state of matter. On the other hand, reduction of an admittedly complex system to a minimal model may also result in a theory that is not of much use in quantitative matching of experimental data. The present reductionist model succeeds in predicting a universal gel volume transition, a point emphasized by Tanaka [10]. It also succeeds in predicting density fluctuations as a feature of the volume phase transition, as observed experimentally [8,9]. How far the theory can be pushed beyond this kind of qualitative descriptive ability is not clear.

The model is based on van der Waals theory, which is usually not extended to ionized systems. However, the model is sufficiently general to apply to polyelectrolyte gels in the absence of added salts, since the counterions can be considered part of a single electroneutral solute. A multicomponent treatment would be required to handle cases like, for example, a cross-linked polyanion network with Na${}^{+}$ counterions along with permeable salt like NaCl. A relatively recent development in the field of free polyelectrolyte solutions is worth mentioning here as of possible interest for polyelectrolyte gels. An effective attractive force between polyelectrolyte chains mediated by condensed counterions, even monovalent counterions, is the probable cause of the observation of loose polyelectrolyte clustering, which should be present in lightly cross-liked swollen polyelectrolyte gels as well. See [11] for background information and many references, and also [12,13,14].

The present model highlights alternatives to rubber as the prototype for the elastic resistance to gel swelling. Anyone who has looked at a table of Poisson ratios for common materials, easily available online, cannot but be struck by the difference between most materials and rubber. Rubber has a Poisson ratio very close to 0.5, while for almost everything else it is in the vicinity of 0.3. The dimensionless Poisson ratio σ for a material in the shape of a cylindrical rod can be written as [15],

(11) $$\sigma =\frac{1}{2}\left(1+\frac{\delta \bar{d}}{\delta \bar{l}}\right)$$

where here $\delta \bar{d}$ is the change in the mass density of the rod material (per unit density) consequent upon an axial length change $\delta \bar{l}$ (per unit length). The maximum value of σ is 1/2, because if were not, a lengthwise compression ($\delta \bar{l}<0$) would result in a decreased density ($\delta \bar{d}<0$), and no material behaves this way. On the other hand, it is easy to see why entropic elasticity results in the value 0.5 for rubber. An extension or compression affects the configurational entropy of the polymer but does not affect molecular structure, so the density change is zero. But for nearly all other materials, say copper for example, an extension of a copper rod increases (slightly) the distance between copper atoms, and the density decreases, although it is admittedly remarkable that the range of σ<0.5 values is so narrow in practice (see online tables). The Poisson ratio for a fully swollen gel has been measured at about 0.25, about the same as for nearly all hard materials and decidedly different from rubber. The polymer network in a swollen gel is stretched; the configurational entropy has been pulled out of the chains. Extension of a swollen gel rod can easily be imagined as affecting the molecular structure, placing strain on the torsion bonds of the polymer, for example, or, since solvent molecules are included in the mass density defining σ, on the hydrogen bonds of an aqueous solvent. Rubber elasticity is not a realistic model for a swollen gel, and although our current attempt in the Langevin function u(r) to find something more appropriate may not be a definitive alternative, it could serve as a marker calling for renewed consideration of this aspect of the gel volume transition.
